# miR-589-3p promoted osteogenic differentiation of periodontal ligament stem cells through targeting ATF1

**DOI:** 10.1186/s13018-022-03000-z

**Published:** 2022-04-10

**Authors:** Fangchuan Shi, Rui He, Jiahao Zhu, Ting Lu, Liangjun Zhong

**Affiliations:** Department of Stomatology, The Affiliated Hospital of Hangzhou Normal University, No. 126, Wenzhou Road, Gongye District, Hangzhou, Zhejiang Province China

**Keywords:** miR-589-3p, Periodontal ligament stem cells, ATF1, Osteogenic differentiation

## Abstract

**Background:**

An increasing number of studies have shown that dysregulated miR-589-3p is associated with multiple diseases. However, the role of miR-589-3p in osteogenic differentiation of periodontal ligament stem cells (PDLSCs) remains unknown. This study aimed to explore the biological function and potential molecular mechanism of miR-589-3p in osteogenic differentiation of PDLSCs.

**Methods:**

GSE159508 was downloaded from Gene Expression Omibus (GEO, http://www.ncbi.nlm.nih.gov/geo/). Differentially expressed miRNAs between osteogenic induction PDLSCs versus non-induction PDLSCs were obtained by R software. miR-589-3p mimic and miR-589-3p inhibitor and corresponding negative control were obtained and to identify the role of miR-589-3p in osteogenic differentiation of PDLSCs. ALP staining and ARS were used to evaluate ALP activity and mineralization, respectively. The targeted binding relationship between miR-589-3p and ATF1 was predicted and verified by target prediction analysis and dual-luciferase assay. Furthermore, the functional mechanism based on miR-589-3p and ATF1 in osteogenic differentiation of PDLSCs was further investigated through rescue experiments.

**Results:**

According to the cut-off criteria with log 2 FC > 1.0 and *P* < 0.05, 514 differentially expressed miRNAs were identified between osteogenic induction and non-induction PDLSCs, including 309 upregulated miRNAs and 205 downregulated miRNAs. Compared with control PDLSCs, miR-589-3p expression level was notably increased in PDLSCs that underwent osteogenic induction. The overexpression of miR-589-3p promoted the cell viability of PDLSCs, while the low expression of miR-589-3p had the opposite effect. The dual luciferase reporter assay verified that ATF1 was proved to be a direct target of miR-589-3p in PDLSCs. And overexpressed miR-589-3p reduced the expression of ATF1. Overexpression of miR-589-3p enhanced the osteogenic capacity of PDLSCs, as demonstrated by increases in ALP activity, matrix mineralization, and RUNX2, OCN and OSX expression. In addition, the rescue experiments confirmed that overexpressed ATF1 restored the effects of overexpressed miR-589-3p on cell proliferation and osteogenic differentiation of PDLSCs.

**Conclusion:**

miR-589-3p could down-regulate the expression of ATF1, thereby promote the proliferation and osteogenic differentiation of PDLSCs. This finding may provide a new therapeutic target for molecular therapy of periodontitis.

**Supplementary Information:**

The online version contains supplementary material available at 10.1186/s13018-022-03000-z.

## Background

The periodontal ligament has stem cells that have the ability to regenerate lost periodontal tissues [1–3]. Orthodontic tooth movement progresses by a combination of periodontal ligament tissue and alveolar bone remodeling processes [4, 5]. Periodontitis is the most common types of diseases that cause bone destruction [6]. Periodontitis, tooth replantation, and repair of bone defects around implants all require periodontal tissue bone regeneration [7].

Periodontal ligament stem cells (PDLSCs) a new population of mesenchymal stem cells (MSCs), exhibited the ability to repair alveolar bone defects in periodontitis [8]. Also, there is need to understand molecular mechanism involved in PDLSCs osteogenic differentiation [9].

MicroRNAs (miRNAs) belong to a class of non-coding RNAs. Mechanically, miRNAs bind to complementary sites on the 3′-untranslated region (3′-UTR) of mRNAs and cause mRNA degradation or translational suppression, thereby inhibiting the expression of target genes [10–12]. MiRNAs are important modulators of normal biological processes, including cell proliferation, differentiation, cell movement, and cell death [13–15].

A growing body of evidence has shown that the dysregulation of miRNA is associated with the osteogenic differentiation of PDLSCs.

For example, miR-22 was elevated in osteogenic PDLSCs, affecting the osteogenic differentiation of PDLSCs through the targeted regulation of HDAC6 [16].

Li et al. [17] observed a notable decrease of miR-24-3p level in osteogenic-differentiated PDLSCs. Further in-depth studies revealed that knockdown of miR-24-3p directly promoted Smad5 and inhibited the osteogenic differentiation of PDLSCs. Xu et al. [18] found that miR-132 was downregulated in osteogenic differentiation of PDLSCs. MiR-132 inhibit PDLSCs osteogenesis via targeting GDF5 and activating NF-κB axis.

miR-589-3p is a multifunction miRNA that participates in many biological processes such as apoptosis, cell proliferation and cellular invasion [19–21]. Cesarini et al. [21] revealed that miR-589-3p/ADAR2 axis controls glioblastoma cell migration and invasion and therefore miR-589-3p may be a therapeutic target for glioblastoma. Guo et al. [19] found that miR‑589‑3p play an important role in breast cancer progression. However, the molecular mechanism of miR-589-3p in osteogenic differentiation of PDLSC has not been reported.

In this study, the downstream target gene of miR-589-3p, namely ATF1, was screened by using bioinformatics methods.

ATF1 may contribute to the growth of lymphomas and transformed lymphocytes [22]. What’s more, ATF1 is required to control gene expression in response to a broad variety of insults, including oxidative stress and cell differentiation [23]. However, the mechanism of ATF1 in osteogenic differentiation of PDLSCs remains to be studied.

Here, the mechanism of miR-589-3p and ATF1 in osteogenic differentiation of PDLSCs was analyzed from the molecular and cellular levels. This study may provide a basis for screening promising biomarkers and potential diagnostic and therapeutic targets for periodontitis.

## Materials and methods

### Bioinformatics analysis

From Gene Expression Omibus (GEO, http://www.ncbi.nlm.nih.gov/geo/), miRNA expression dada (GSE159508) was downloaded. According to differential analysis, miRNA data included 3 osteogenic induction PDLSCs and 3 non-induction PDLSCs. With |logFC|> 2 and *P* value < 0.05 as standards, differential miRNAs were obtained. Downstream target mRNAs with targeted binding sites of miR-589-3p were predicted through the Targetscan, miRDB and miRanda databases. To identify target gene that may be regulated by miR-589-3p, we generated a venn diagram illustrating the intersection between the target genes of Targetscan, miRDB and miRanda through Draw Venn Diagram online tool (http://bioinformatics.psb.ugent.be/webtools/Venn/).

Gene ontology (GO) terms and Kyoto Encyclopedia of Genes and Genomes (KEGG) pathway of the target genes of miR-589-3p were performed using the Database for Annotation, Visualization and Integrated Discovery (DAVID, https://david.ncifcrf.gov/) [24]. GO terms including biological process (BP), cellular component (CC) and molecular function (MF) [25].

### Cell culture

Human PDLSCs were all purchased from BeNa culture collection as previously described [26]. PDLSCs were incubated in Dulbecco’s modified Eagle medium (DMEM) (Sigma, USA) containing 10% fetal bovine serum (FBS) (Hyclone, GE Healthcare Life Sciences, Logan, UT, USA) in a constant temperature incubator (37 °C) with 5% CO_2_.

### Cell transfection

miR-589-3p mimic, miR-589-3p inhibitor, oe-ATF1, sh-ATF1, mimic NC, inhibitor NC, oe-NC and sh-NC, obtained from Sangon Biotech (Shanghai, China), were transfected into cell line PDLSCs with the help of Lipofectamine 2000 (Thermo Fisher Scientific, Inc., USA) according to the manufacturer’s instructions. After 4 h, the medium without FBS was replaced with a complete medium containing 20% FBS, and the cells were cultured under the corresponding culture conditions for 48 h for later use.

### ALP and ARS staining

ALP staining was monitored using an ALP staining kit according to the manufacturer’s protocol. Briefly, PDLSCs were fixed in 4% paraformaldehyde for 10 min and washed with PBS for three times. Then, PDLSCs were stained by BCIP/NBT Alkaline Phosphatase Color Development Kit (Beyotime, Shanghai, China) for 30 min. Then, distilled water was added to abort the reaction. A microscope (CX41, OLYMPUS Optical Co., Ltd., Tokyo, Japan) was used to observe and photograph. Mineral deposition was monitored using an ARS staining kit (Solarbio, Beijing, People’s Republic of China) according to the manufacturer’s protocol. Briefly, PDLSCs were fixed in 4% paraformaldehyde for 10 min and washed with PBS for three times. Then, PDLSCs were stained by 40 mM Alizarin Red S (ARS, Sigma) for 30 min. Then, distilled water was added to abort the reaction. A microscope (CX41, OLYMPUS Optical Co., Ltd., Tokyo, Japan) was used to observe and photograph.

### qRT-PCR

Total RNA was extracted from cells using Trizol Reagent (Invitrogen, USA), and then reversely transcribed into complementary DNA (cDNA) using PrimeScript RT Reagent Kit (Takara, RR047A, China). Primer sequences are shown in Table 1. qRT-PCR system was applied according to the manufacturer’s instructions provided by SYBR Prime Script RT-PCR Kits (Takara). The reaction parameters included pre-denaturation at 95 °C for 10 min, followed by 35 cycles of denaturation at 95 °C for 10 s, annealing at 57 °C for 30 s and extension at 72 °C for 30 s. U6 and GAPDH were employed as internal parameters for miR-129-5p and TRIP13, respectively. The relative expression of miR-589-3p and ATF1 mRNA were calculated by 2^−ΔΔCt^ method.

**Table 1:** Top twenty differentially expressed miRNAs

miRNAs	logFC	AveExpr	*t*	*P* value	adj. *P* Val	*B*
hsa-miR-4754	− 3.35008	− 5.75931	− 8.42111	8.72E− 05	0.050012	2.016328
hsa-miR-4513	− 4.42802	− 4.27425	− 9.41023	0.000116	0.050012	1.637959
hsa-miR-219b-3p	3.62806	− 3.10675	8.451345	0.000205	0.050012	1.205433
hsa-miR-5011-3p	3.62806	3.203451	8.451345	0.000205	0.050012	1.205433
hsa-miR-5188	− 2.87531	− 6.2763	− 7.22424	0.000221	0.050012	1.198741
hsa-miR-5700	3.725116	− 3.20514	8.376855	0.000215	0.050012	1.168755
hsa-miR-4768-3p	− 3.58104	− 4.26587	− 8.20705	0.000239	0.050012	1.083349
hsa-miR-370-3p	− 2.46344	− 6.35636	− 6.95195	0.000278	0.050012	0.990432
hsa-miR-650	− 2.85137	− 5.91361	− 6.87311	0.000297	0.050012	0.928424
hsa-miR-589-3p	3.876845	3.25121	7.720135	0.00033	0.050012	0.823905
hsa-miR-96-3p	3.876845	5.98767	7.720135	0.00033	0.050012	0.823905
hsa-miR-1257	− 2.52899	− 6.32359	− 6.73445	0.000336	0.050012	0.817493
hsa-miR-758-3p	2.611378	− 2.97964	6.696672	0.000347	0.050012	0.786842
hsa-miR-2909	3.919067	2.89692	7.479284	0.000389	0.050012	0.687104
hsa-miR-337-3p	2.259118	0.005956	6.557568	0.000392	0.050012	0.672397
hsa-miR-3713	2.92615	− 5.33822	6.521763	0.000405	0.050012	0.642529
hsa-miR-330-3p	− 3.22127	5.88691	− 7.32923	0.000432	0.050012	0.59887
hsa-miR-4670-5p	3.394881	6.66149	7.237235	0.000461	0.050012	0.543599
hsa-miR-4781-5p	3.394881	8.87918	7.237235	0.000461	0.050012	0.543599
hsa-miR-5189-5p	− 3.11412	− 6.42764	− 7.18391	0.000479	0.050012	0.511141

### Western blot

Radioimmunoprecipitation assay (RIPA) reagent (Beyotime, China) was used to extract total proteins 48 h after cell transfection. Protein samples were loaded and isolated by 10% sodium dodecyl sulfate polyacrylamide gel electrophoresis (SDS-PAGE), then transferred to polyvinylidene fluoride (PVDF) membrane. The membrane was sealed in 5% skimmed milk in Tris buffered saline-Tween (TBST) buffer at room temperature for 1 h. The membrane was washed with TBST and incubated with anti-TRIP13 (1:500, ab64964, abcam, UK) and anti-GAPDH (1:10,000, ab181602, abcam, UK). Then, the membrane was washed again with Na2SO3 3 times. Next, the membrane was incubated with horseradish peroxidase (HRP) conjugated second antibody (Beyotime) at room temperature for 1–2 h. The membrane was washed with TBST buffer solution 3 times, 10 min each. Finally, images were developed with an optical luminescence instrument (GE, USA) and photographed.

### Dual-luciferase reporter assay

Target fragment was inserted into the luciferase vector pmiRGLO (Promega, USA) using T4 DNA ligase to construct luciferase reporter plasmids wild-type (WT)-ATF1 and mutant (MUT)-ATF1, with the following corresponding sequences on 3′UTR: WT-ATF1: 5′-CGUUUGAUUUAGUGCAAAAAU-3′; MUT-ATF1: 5′-CUUUCGGUUGGAUUACGCAAA-3′. For dual-luciferase reporter gene assay, WT-ATF1 or MUT-ATF1 was co-transfected with mimic NC or miR-589-3p mimic into cells. After 48 h, luciferase activity was measured using the Dual-luciferase Reporter Assay System (Promega, USA) according to the manufacturer’s requirements.

### Data analysis

All data were processed using GraphPad Prism 8 (GraphPad Software Inc., USA) statistical software. Measurement data were expressed in the form of mean ± standard deviation. The comparison between two groups was performed by *t* test. *P* < 0.05 indicates a statistically significant difference.

## Results

### Bioinformatic analysis results

**Fig. 1:**
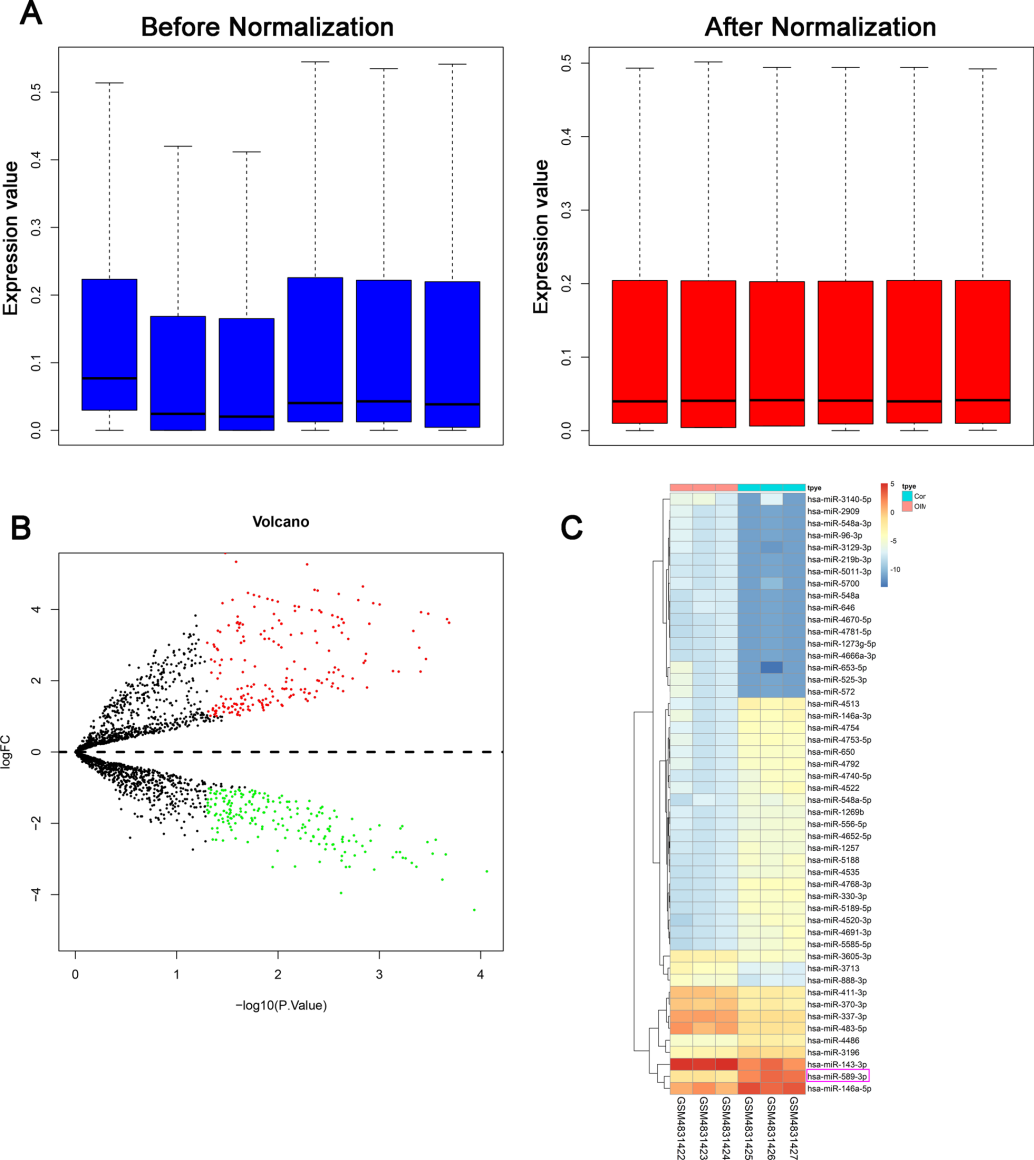
Bioinformatic analysis of the differentially expressed miRNAs between non-induction and osteogenic induction PDLSCs. **A** expression value before and after normalization; **B** Heatmap of the differentially expressed miRNAs between control and osteogenic induction PDLSCs; Red dots, upregulated miRNAs, Green dots, downregulate dots and black dots, nondifferentially expressed miRNAs. **C** Volcano plot the differentially expressed miRNAs between control and osteogenic induction PDLSCs. Red represented upregulated miRNAs, Green represented downregulate miRNAs

The log-expression values were normalized and the expression values in the samples were all identical (Fig. 1A). Among the miRNAs, 513 differentially expressed miRNAs with *P* ≤ 0.05 and log FC > 1 were identified.

According to the cut-off criteria with log 2 FC > 1.0 and *P* < 0.05, 514 differentially expressed miRNAs were identified between osteogenic induction and non-induction PDLSCs, including 309 upregulated miRNAs and 205 downregulated miRNAs.

The differentially expressed miRNAs were illustrated in the volcano plot (Fig. 1B) and heatmap (Fig. 1C). Top twenty miRNAs can be seen in Table 1.

miR-589-3p was the most upregulated miRNAs and thus selected for further study.

**Fig. 2:**
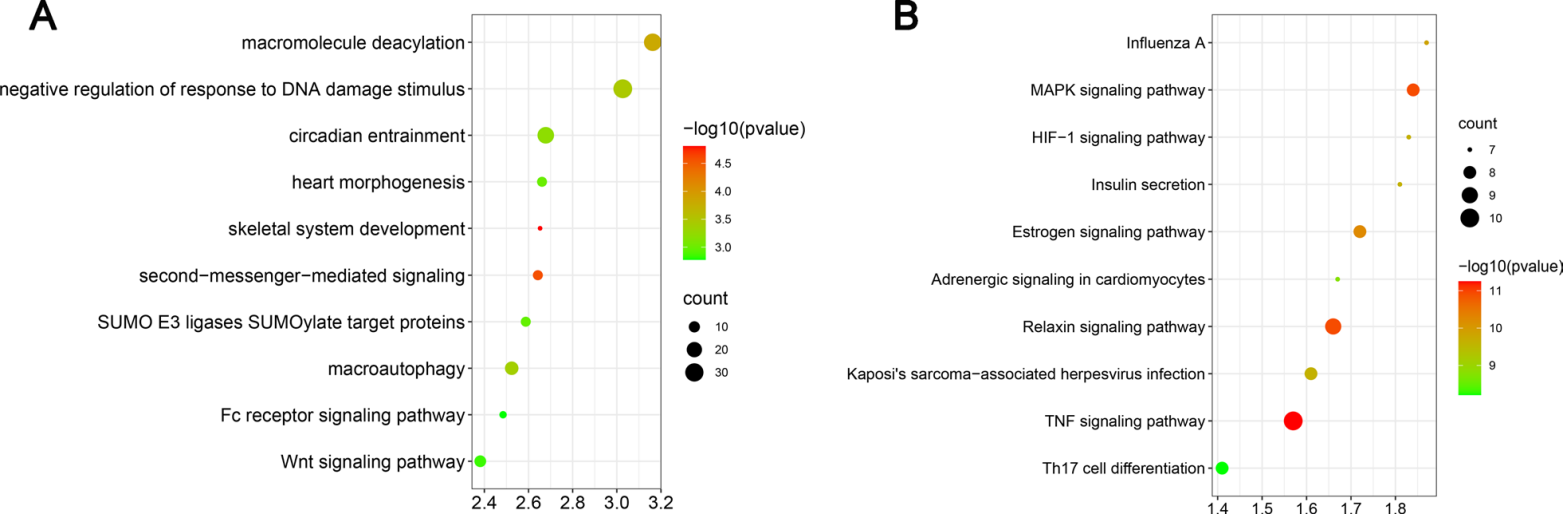
Biological function of the miR-589-3p target genes. **A** Gene ontology of the target genes of miR-589-3p, the node color reflects *P* value (− log10(*P* value)): the bigger the − log10(*P* value) value, the darker the node color is. **B** KEGG pathway of the target genes of miR-589-3p. The node color reflects *P* value (− log10(*P* value)): the bigger the − log10(*P* value) value, the darker the node color is. The size of circle represents gene count

Functional annotation using Gene Ontology (GO) enrichment analysis was performed to determine the main functions of putative target genes of miR-589-3p. Top ten GO terms were as follows: macromolecule diacylation, negative regulation of response to DNA damage stimulus, circadian entrainment, heart morphogenesis, skeletal system development, second-messenger-mediated signaling, SUMO E3 ligases SUMOylate target proteins, macroautophagy, Fc receptor signaling pathway and Wnt signaling pathway (Fig. 2A).

Top ten KEGG pathways were as follows: Influenza A, MAPK signaling pathway, HIF-1 signaling pathway, Insulin secretion, Estrogen signaling pathway, Adrenergic signaling in cardiomyocytes, Relaxin signaling pathway, Kaposi’s sarcoma-associated herpesvirus infection, TNF signaling pathway and Th17 cell differentiation (Fig. 2B).

### Characterization of PDLSCs

**Fig. 3:**
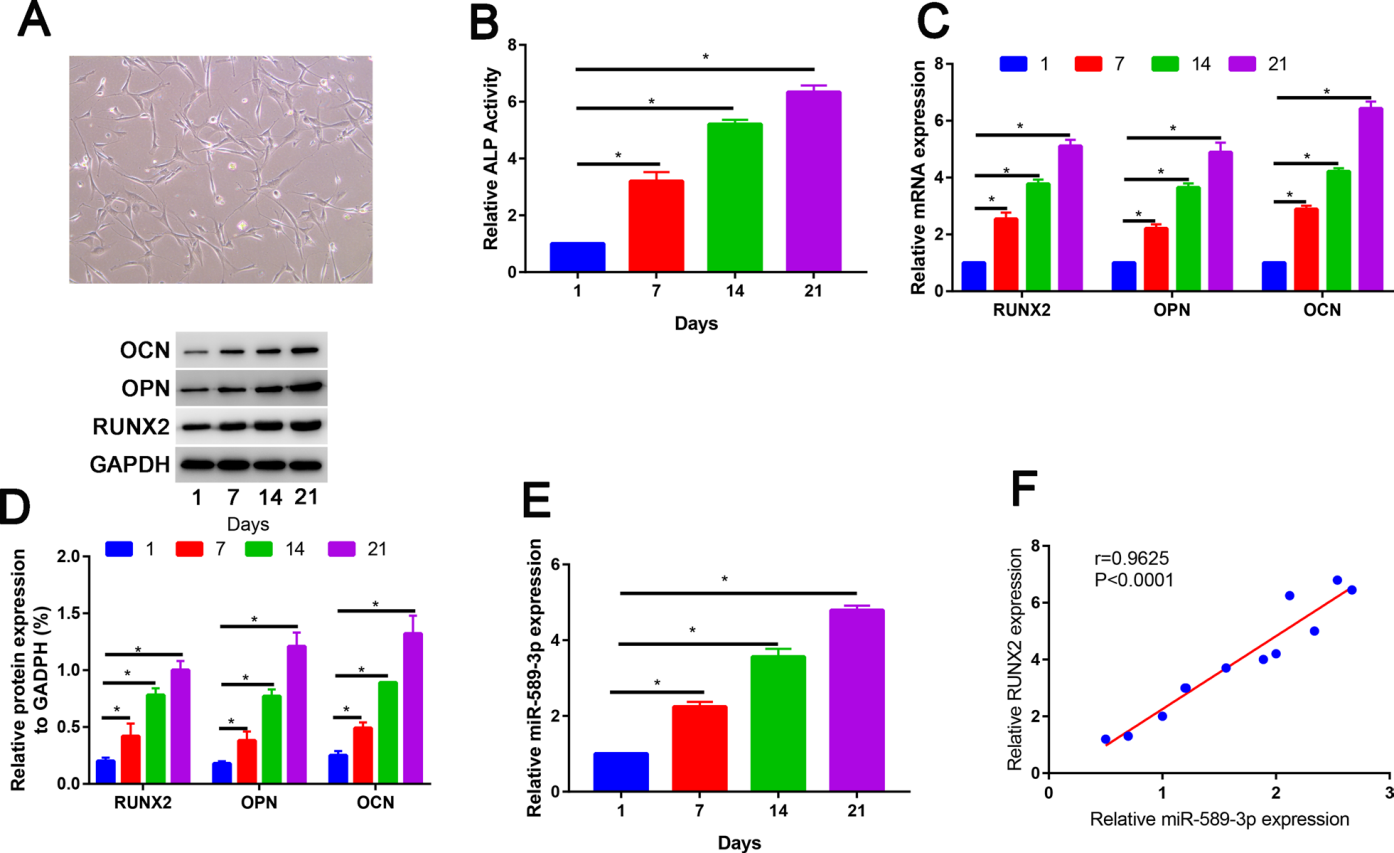
The expression of miR-589-3p in PDLSCs during osteogenic differentiation. **A** PDLSCs were observed under a light microscope; **B** ALP activity of PDLSCs at the indicated days (1, 7, 14 and 21 days) after the osteogenic induction; **C** Relative RUXN2, OPN and OCN mRNA expression of PDLSCs at the indicated days (1, 7, 14 and 21 days) after the osteogenic induction; **D** Relative RUXN2, OPN and OCN protein expression of PDLSCs at the indicated days (1, 7, 14 and 21 days) after the osteogenic induction; **E** Relative miR-589-3p mRNA expression of PDLSCs at the indicated days (1, 7, 14 and 21 days) after the osteogenic induction; **F** Spearman correlation analysis among miR-589-3p expression and RUNX2 expression. *P < 0.05

PDLSCs were spindle-shaped and fibroblast-like and exhibited a characteristic spindle-shaped appearance (Fig. 3A).

As the time of osteogenic induction time prolong, the RUNX2, OCN and OSX mRNA expression was increased (Fig. 3B). In accordance with increased RUNX2, OCN and OSX mRNA expression, ALP activity increased in the presence of osteogenic medium (Fig. 3C). Western blot results were in accordance with qRT-PCR results (Fig. 3D). miR-589-3p expression was significantly increased after osteogenic induction (Fig. 3E). Moreover, the miR-589-3p expression was positively correlated with the RUNX2 expression (Fig. 3F).

### miR-589-3p promotes the proliferation of PDLSCs in vitro

**Fig. 4:**
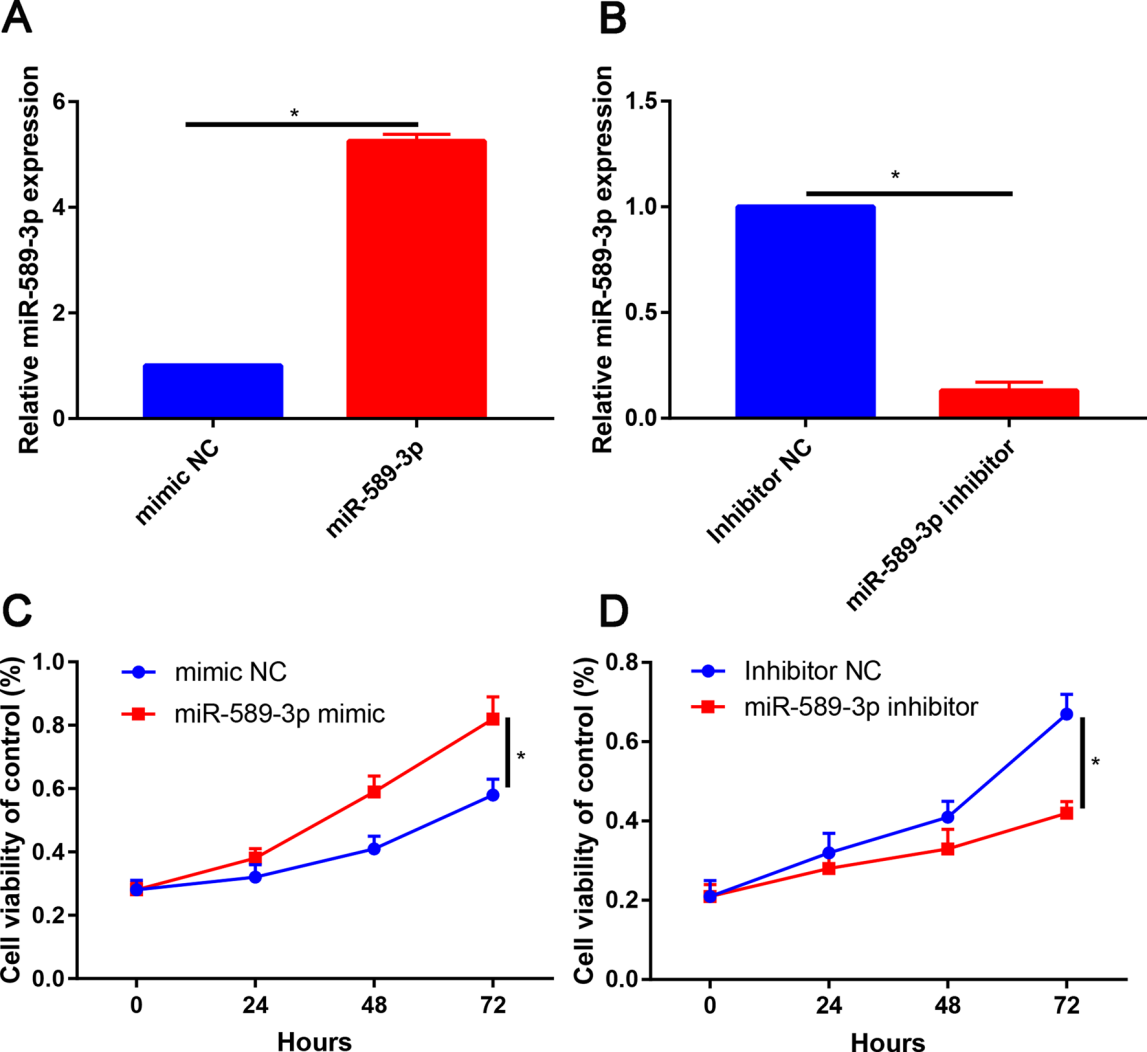
miR-589-3p promoted the cell viability of PDLSCs. **A** Relative miR-589-3p expression in mimic NC and miR-589-3p groups; **B** Relative miR-589-3p expression in inhibitor NC and miR-589-3p inhibitor groups; **C** Cell viability of PDLSCs between mimic NC and miR-589-3p groups at 0, 24, 48 and 72 h; **D** Cell viability of PDLSCs between inhibitor NC and miR-589-3p inhibitor groups at 0, 24, 48 and 72 h. *P < 0.05

PDLSCs were transfected with miR-589-3p mimic, miR-589-3p inhibitor and corresponding negative control, transfection efficacy was verified by qRT-PCR. It was noted that miR-589-3p mimic transfection significantly increased miR-589-3p level in PDLSCs compared with the mimic NC (Fig. 4A), whereas miR-589-3p inhibitor transfection significantly decreased the miR-589-3p expression level (Fig. 4B).

CCK-8 assay was used to determine proliferation of PDLSCs, and the results showed that overexpression of miR-589-3p promoted PDLSCs proliferation, whereas knockdown of miR-589-3p suppressed PDLSCs proliferation (Fig. 4C, D, *P*  < 0.05).

## miR-589-3p facilitate the osteogenic differentiation of PDLSCs in vitro

To elucidate the effect of miR-589-3p on the efficiency of PDLSC differentiation, the transfected hPDLSCs were induced to differentiate along osteogenic lineages for 7 days and 21 days, followed by measuring the ALP and ARS staining, 7 days and 21 days after the induction respectively.

**Fig. 5:**
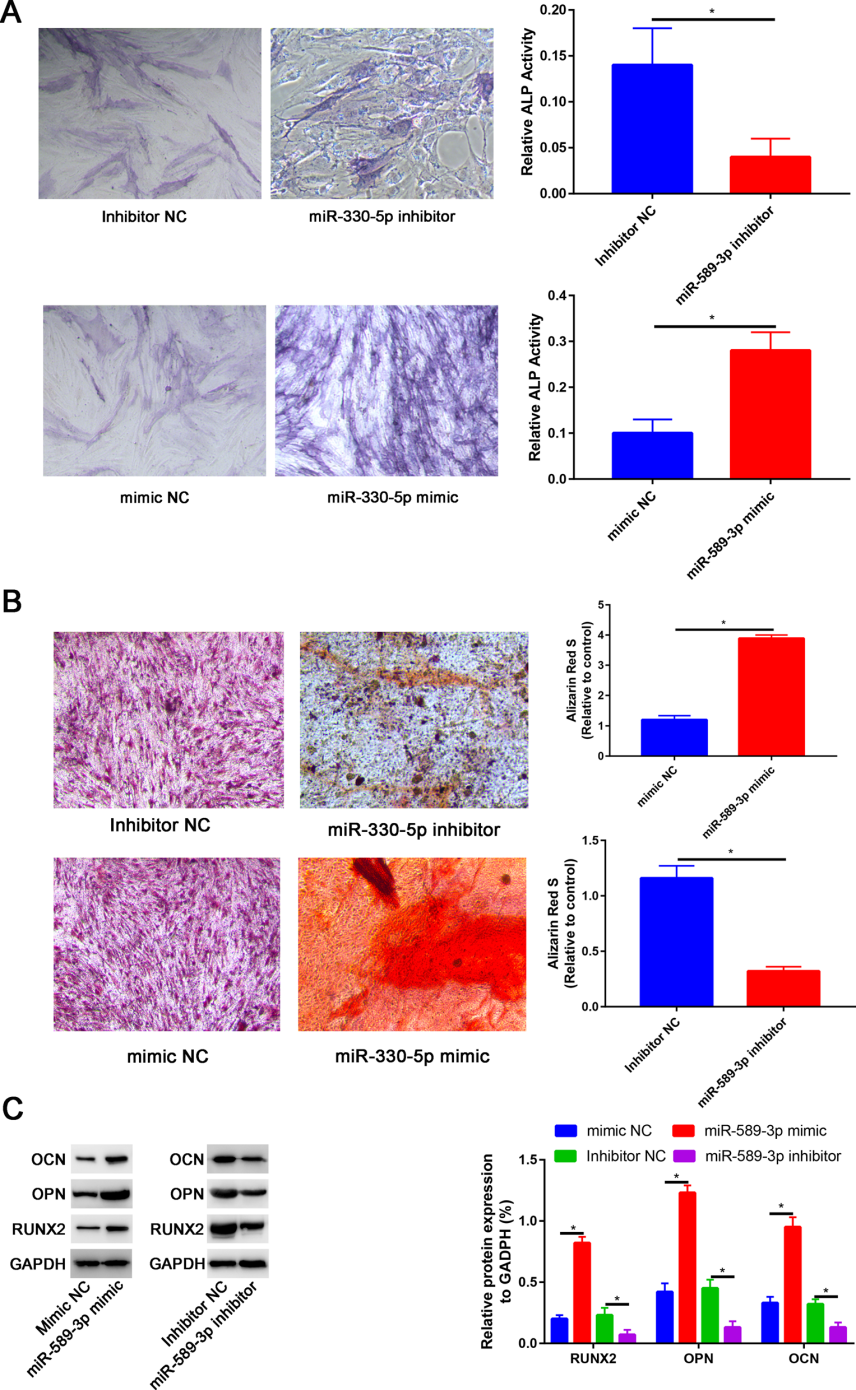
miR-589-3p promoted osteogenic differentiation of PDLSCs. **A** Images of ALP staining in the inhibitor NC, miR-589-3p inhibitor, mimic NC and miR-589-3p mimic groups; **B** Images of Alizarin Red S staining in the inhibitor NC, miR-589-3p inhibitor, mimic NC and miR-589-3p mimic groups; **C** Western blot assay to assess the RUNX2, OPN and OCN expression in the inhibitor NC, miR-589-3p inhibitor, mimic NC and miR-589-3p mimic groups. *P < 0.05

The relative ALP activity of the miR-589-3p mimic group was dramatically higher than that of the mimic NC group, and this result was statistically significant (Fig. 5A, *P* < 0.001).

21 days after osteogenic induction, Alizarin Red S staining and calcium quantitation assays were performed, which revealed an increased extent of osteoblastic mineralization in the miR-589-3p mimic group in comparison to mimic NC group (Fig. 5B).

mRNA expression levels of the markers RUNX2, OPN and OCN were elevated at osteogenesis day 7 by miR-589-3p mimic whereas they were impaired by miR-589-3p inhibitor when compared to corresponding negative control (*P* < 0.05).

By contrast, miR-589-3p inhibitor could reduce ALP activity level and calcium deposition, and decrease the expression levels of RUNX2, OPN and OCN of PDLSCs in compared with the inhibitor negative control (Fig. 5C, *P* < 0.05).

### miR-589-3p directly targets ATF1 in PDLSCs

To reveal the molecular mechanism by which miR-589-3p regulates osteogenic differentiation of PDLSCs, TargetScan, miRanda and miRDB databases were used to predict potential targets of miR-589-3p.

The venn diagram represents the genes in the three individual databases and overlapping 34 putative target genes identified by integrated analysis of the three databases (TargetScan, miRanda and miRDB, Additional file 1: S1).

**Fig. 6:**
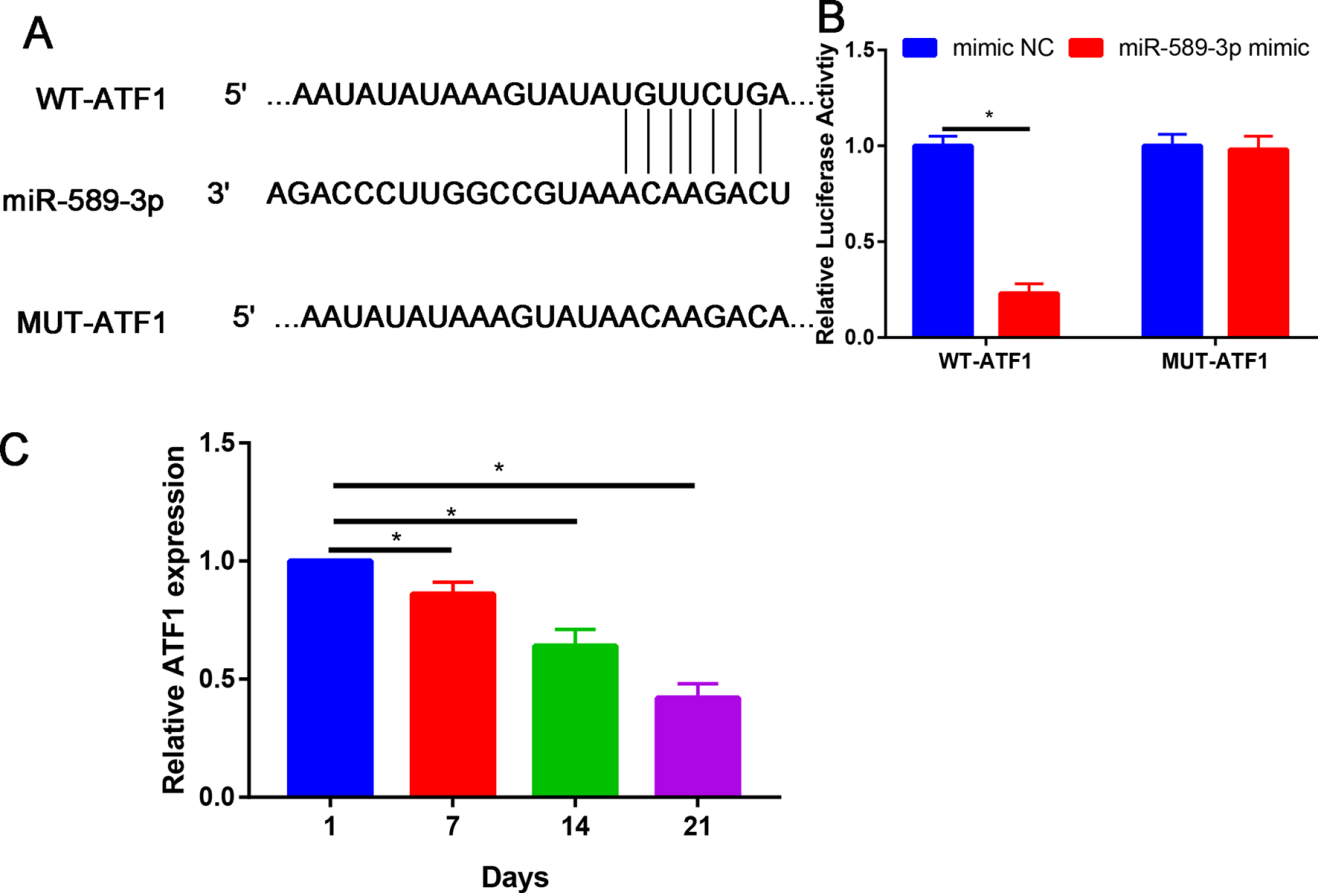
miR-589-3p directly target to ATF1 3′UTR. **A** Putative binding sites between miR-589-3p and ATF1; **B** Luciferase reporter assay showed that miR-589-3p mimic transfection suppressed the relative luciferase activity of the ATF1-Wt reporter in PGLSCs; **C** Relative ATF1 expression in inhibitor NC, miR-589-3p inhibitor, mimic NC and miR-589-3p mimic groups. *P < 0.05

ATF1 3′UTR sequence contained the binding sites for miR-589-3p (Fig. 6A), implying that ATF1 might be a downstream target of miR-589-3p.

The luciferase activity assay revealed the miR-589-3p mimic suppressed ATF1 3′-UTR wild-type (WT) luciferase activity, whereas it had no effect on ATF1 3′-UTR mutant (Mut) luciferase activity compared with control in PDLSCs (Fig. 6B).

In contrast to miR-589-3p expression, Similar to BM-MSCs, the expression of ATF1 during the process of osteogenic differentiation of PDLSCs was reduced in a time-dependent manner (Fig. 6C).

### Overexpression ATF1 reversed the effects of miR-589-3p on proliferation and osteogenic differentiation of PDLSCs

In order to validate whether miR-589-3p promoted osteogenic differentiation of PDLSCs by inhibiting ATF1, the rescue experiments were performed by co-transfection with miR-589-3p mimic and ATF1.

**Fig. 7:**
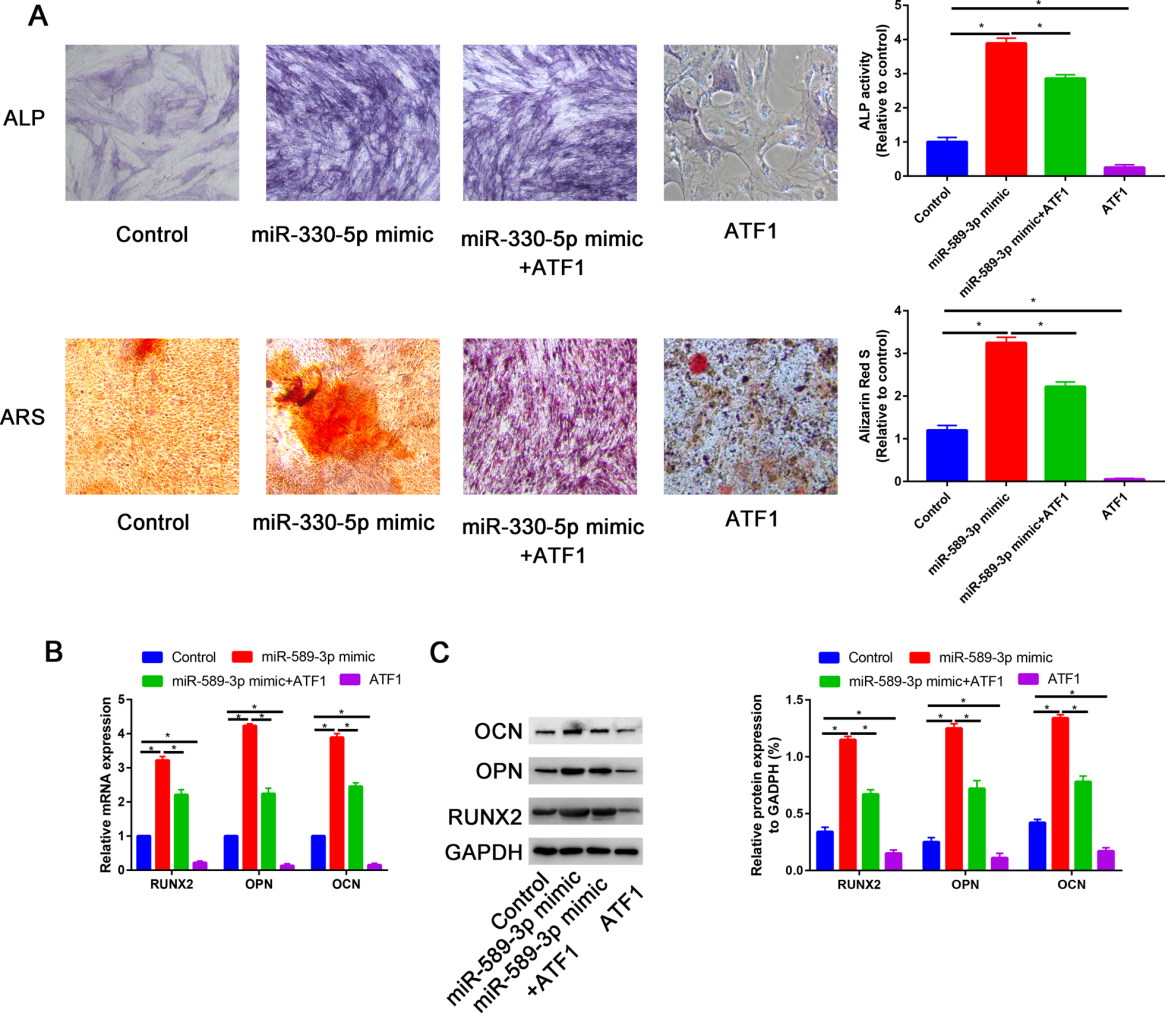
Overexpression ATF1 partially reversed the effects of miR-589-3p-overexpression on PDLSCs. **A** Images of ALP and Alizarin Red S staining in control, miR-589-3p mimic, miR-589-3p mimic + ATF1 and ATF1 groups; **B** Relative RUNX2, OPN and OCN mRNA expression in control, miR-589-3p mimic, miR-589-3p mimic + ATF1 and ATF1 groups; **C** Relative RUNX2, OPN and OCN protein expression in control, miR-589-3p mimic, miR-589-3p mimic + ATF1 and ATF1 groups. *P < 0.05.

Compared with control group, better ALP activity and mineralized nodule formation was found in miR-589-3p group, but partially reversed by co-transfection with ATF1 overexpression plasmid. Meanwhile, ATF1 overexpression alone could also delay the osteogenic differentiation process (Fig. 7A). Real-time PCR and western blot assays further confirmed that the upregulation of RUNX2, OPN and OCN induced by miR-589-3p could be partially blockaded by overexpression of ATF1. Moreover, overexpression of ATF1 could also decrease the RUNX2, OPN and OCN expression (Fig. 7B, C).

## Discussion

In this study, we firstly identified the differentially expressed miRNAs between osteogenic induction and non-osteogenic induction PDLSCs through bioinformatic analysis. miR-153-3p mimic or ATF1 suppression promoted the osteogenic differentiation of PDLSCs, as demonstrated by increases in ALP activity and matrix mineralization. Further studies revealed that miR-589-3p promoted osteogenic differentiation of PDLSCs by sponging ATF1.

Among these altered miRNAs, miR-589-3p was chosen for further study for two reasons. First, miR-589-3p showed a significant fold change among the upregulated miRNAs. Also, this is the first report of altered miR-589-3p expression in osteogenic differentiation of PDLSCs. miR-589-3p upregulation promoted the osteogenic differentiation of PDLSCs, as demonstrated by increases in ALP activity, matrix mineralization, and ALP, Runx2, and OPN expression.

MicroRNA (miRNA) has been reported to become novel therapeutic targets for skeletal related diseases.

Some miRNAs are differentially expressed in stem cells, and have significant effects on the osteogenic differentiation of stem cells. Lu et al. [20] identified the role of miR-589-3p in human lumbar disc degeneration and its potential mechanism. They found that miR-589-3p was significantly upregulated in lumbar disc degeneration patients. Following dual-luciferase reporter assay, Smad was demonstrated to be a target gene for miR-589-3p.

Furthermore, ATF1, which is a target gene of miR-589-3p, decreased osteogenic differentiation of PDLSCs; however, these effects were partially reversed by miR-589-3p mimic.

ATF1 has been shown to play an important role in cell proliferation, differentiation and apoptosis [27]. ATF1, as a regulator, also promoting sexual differentiation and entry into the stationary phase in S. pombe [28]. AtfA and Atf1 are quite highly conserved and that they are involved in multiple cellular processes [28]. In this study, we found that overexpression of ATF1 partially reversed the promotion effects of miR-589-3p on osteogenic differentiation of PDLSCs. Moreover, luciferase activity verified that miR-589-3p could significantly inhibited the luciferase activity of wild-type ATF1, but it failed to suppress luciferase activity of mutated one.

Limitation of this study can be listed as follows: (1) in vivo study was lack and thus should be further study in future studies; (2) following signaling pathway should be further explored the mechanism of miR-589-3p.

## Conclusion

In conclusion, this study suggested that miR-589-3p promotes osteogenic differentiation of PDLSCs by targeting ATF1. The results of the present study demonstrated that the miR-589-3p/ATF1 interaction network may serve as a potential regulatory mechanism underlying PDLSCs osteogenesis.

## Supplementary Information

**Additional file 1: S1.** Venn diagram revealed the overlapped miRNAs in Targetscan, miRanda and miRDB databases.

## Data Availability

All the data will be available upon motivated request to the corresponding author of the present paper.

## Abbreviations

PDLSCs: Periodontal ligament stem cells
GEO: Gene Expression Omibus
MSCs: Mesenchymal stem cells
3′-UTR: 3′-Untranslated region
GO: Gene ontology
DAVID: Database for Annotation, Visualization and Integrated Discovery
BP: Biological process
CC: Cellular component
MF: Molecular function
DMEM: Dulbecco’s modified Eagle medium
FBS: Fetal bovine serum
PVDF: Polyvinylidene fluoride
TBST: Tris buffered saline-Tween
