# Supplementary figures and images for: miR-589-3p promoted osteogenic differentiation of periodontal ligament stem cells through targeting ATF1
Source: J Orthop Surg Res. 2022 Apr 10;17:221. doi: 10.1186/s13018-022-03000-z (PMC8996605; doi:10.1186/s13018-022-03000-z)

Targetscan

miRDB

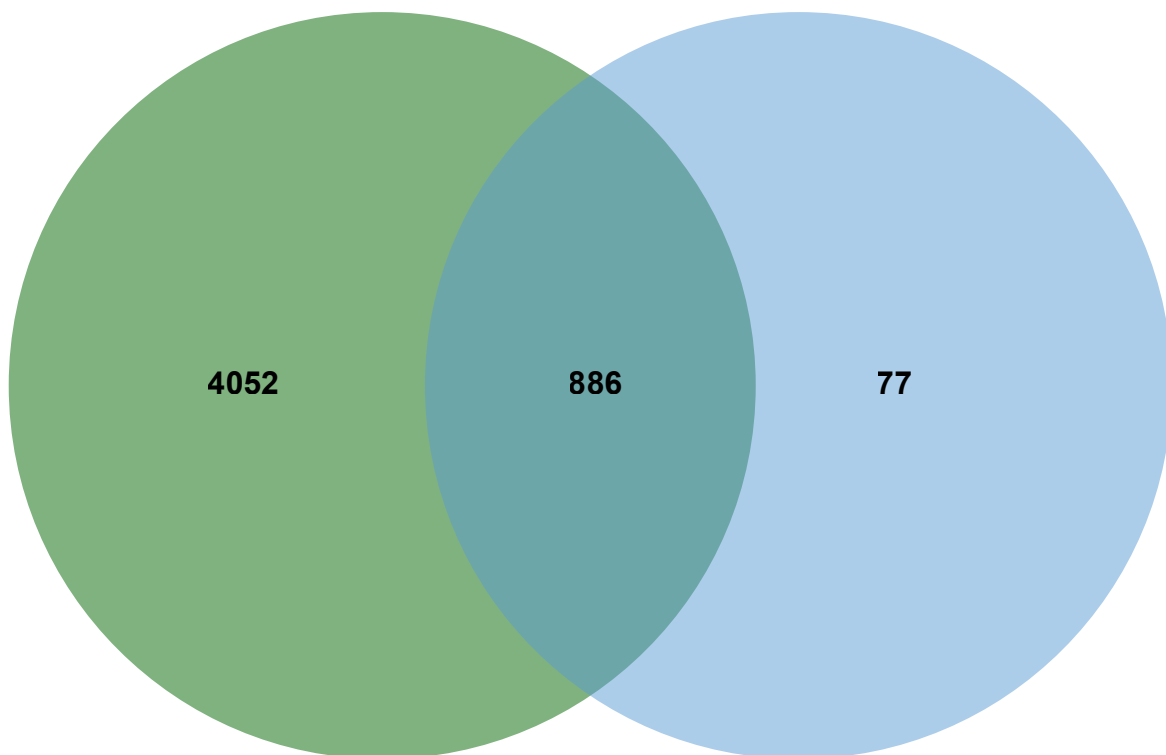

*Size of each list*

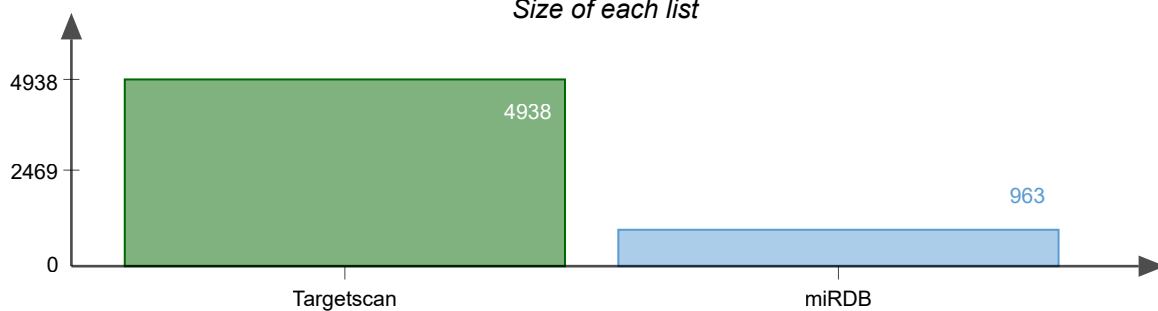

*Number of elements: specific (1) or shared by 2, 3, ... lists*

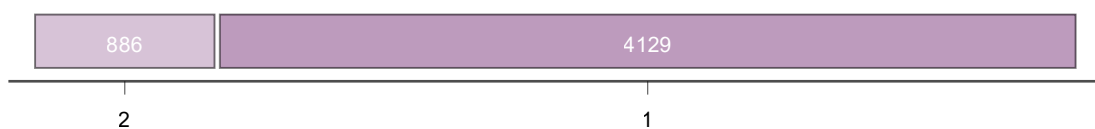

Supplement: Supplementary file 1 — Additional file 1: S1. Venn diagram revealed the overlapped miRNAs in Targetscan, miRanda and miRDB databases. [file 13018_2022_3000_MOESM1_ESM.pdf]
